# Unique Environmental Effects on Physical Activity Participation: A Twin Study

**DOI:** 10.1371/journal.pone.0002019

**Published:** 2008-04-16

**Authors:** Glen E. Duncan, Jack Goldberg, Carolyn Noonan, Anne Vernez Moudon, Philip Hurvitz, Dedra Buchwald

**Affiliations:** 1 Department of Epidemiology, University of Washington, Seattle, Washington, United States of America; 2 Vietnam Era Twin Registry, Seattle VA Epidemiologic Research and Education Center, Seattle, Washington, United States of America; 3 Department of Medicine, University of Washington, University of Washington Twin Registry, Seattle, Washington, United States of America; 4 Department of Urban Design and Planning, University of Washington, Seattle, Washington, United States of America; The Royal College of Physicians and Surgeons of Canada, Canada

## Abstract

**Background:**

The health benefits of regular physical activity are well established. However, the relative contribution of heritable and environmental factors to physical activity participation remains controversial. Using a cut-point of 60 minutes of total activity per week, data from the GenomEUtwin project revealed consistent genetic influence on physical activity participation in 37,051 twin pairs from seven countries. We hypothesized that the heritability of physical activity participation would be attenuated using the CDC/ACSM recommended minimum threshold of 150 minutes of moderate intensity activity per week.

**Methods:**

Data were obtained from 1,389 twin pairs from the community-based University of Washington Twin Registry. Twin similarity in physical activity participation using both cut-points was analyzed using tetrachoric correlations and structural equation modeling in all same-sex pairs.

**Results:**

Correlations were higher in monozygotic (r_MZ_ = 0.43, 95% CI = 0.33–0.54) than dizygotic pairs (r_DZ_ = 0.30, 95% CI = 0.12–0.47) using the 60 minute cut-point. However, differences were attenuated using the 150 minute standard (r_MZ_ = 0.30, 95% CI = 0.20–0.40; r_DZ_ = 0.25, 95% CI = 0.07–0.42). Using the lower cut-point, the best fitting model of twin resemblance only included additive genetics and unique environment, with a heritability of 45%. In contrast, using the higher threshold, the best fitting model included the common and unique environment, with the unique environment contributing 72% of the variance.

**Conclusion:**

Unique environment factors provide the strongest influence on physical activity participation at levels recommended for health benefits.

## Introduction

The health benefits of regularly performed physical activity are well established and include weight control, improved cardiorespiratory fitness, and decreased risk for chronic diseases such as cardiovascular disease, type 2 diabetes, and some forms of cancer [1]–[5]. Despite the recognized health benefits of an active lifestyle, the majority of the U.S. population, and that of many other industrialized nations, does not engage in physical activity levels consistent with recommendations for achieving health benefits [6]–[9]. Thus, identifying the barriers to and facilitators of regular participation in physical activity is critical for health promotion.

An ecological model proposes that physical activity behaviors are influenced by many interacting factors that range from biology to policy [10]. However, the relative contribution of genetic and shared and nonshared environmental factors on physical activity participation remains controversial. Most studies indicate moderate to strong genetic effects on physical activity participation [11]–[18]. The largest study, which pooled data from 7 European twin registries and 37,051 twin pairs (GenomEUtwin project) revealed a consistent genetic influence on physical activity participation, with heritability estimates ranging between 48–71% [15]. In this study, researchers used a cut-point of 60 minutes of activity per week, eliciting a minimum intensity of four metabolic equivalents (4 METs; where 1 MET = 3.5 mL·min^−1^·kg^−1^), to categorize twins as exercisers and non-exercisers. This cut-point was chosen based on the need to provide a dichotomy that would be reasonably comparable across different physical activity instruments used in the various countries [15], rather than an empirically derived threshold, such as the Centers for Disease Control and Prevention and American College of Sports Medicine (CDC/ACSM) [19], [20] recommended minimum threshold of 150 minutes of moderate intensity activity per week, that is associated with health benefits. Furthermore, most studies examining the heritability of physical activity were conducted outside of the U.S., including Australia, Denmark, Finland, Norway, The Netherlands, Sweden and United Kingdom participating in the GenomEUtwin project. This is an important consideration because of environmental, and possibly genetic, differences that exist across these countries.

The purpose of this study was to examine the heritability of physical activity participation in adult U.S. twins using two different cut-points. We hypothesized that the heritability of physical activity participation would be attenuated in twins classified as exercisers and non-exercisers using the empirically-derived CDC/ACSM [19], [20] recommended minimum threshold of 150 minutes of moderate intensity activity per week, compared to the 60 minute per week threshold used in the GenomEUtwin project.

## Methods

### Subjects

The study sample consisted of 1,389 same-sex twin pairs from the University of Washington Twin Registry (UWTR). The UWTR is a community-based sample of adult twin pairs assembled from Washington State Department of Licensing records. In Washington State, all new drivers license and identification card applicants are asked if they are a member of a twin or higher multiple birth. Since June 1999, data on all new twin applicants has been transmitted to the UWTR from the Department of Licensing. To date, there are 2,412 twin pairs in the registry, and in approximately 58% of pairs, both twins are living in Washington State.

### Data collection

All twins were mailed a brief survey that included items on zygosity, socio-demographics, height and weight, general health and common medical conditions, and lifestyle behaviors. A small incentive for completing the survey was included in the mailing, and written informed consent was provided by all twins. These procedures are approved by the university's institutional review board.

### Measures

Zygosity was determined using standard questions on childhood similarity that correctly assign zygosity at least 95% of the time [21], [22]. The UWTR survey asks respondents how many times per week they exercise moderately for at least 30 minutes and vigorously for at least 20 minutes. We constructed a physical activity measure by summing the reported number of moderate and vigorous blocks of activity to estimate the total minutes per week of moderate-to-vigorous activity. We then created two separate dichotomous variables based on different cut points for total physical activity: at least 60 minutes per week, conforming to that used in the GenomEUtwin collaborative study and at least 150 minutes per week, conforming to the level recommended by the CDC/ACSM to achieve health benefits.

### Statistical analysis

Descriptive statistics were calculated on select demographic characteristics and presented as means and standard deviations for continuous variables and percents for categorical variables. Statistical inference for demographic comparisons was made using generalized estimating equations, which adjust standard errors for the correlation within twin pairs.

We computed the within-pair tetrachoric correlations and 95% confidence intervals for each binary activity measure separately in monozygotic (MZ) and dizygotic (DZ) twins. We then used structural equation modeling to estimate the genetic and nongenetic contribution to physical activity [23]. Models were fit to the pattern of twin correlations to estimate the amount of phenotypic variance due to additive genetic (A), common environmental (C), and unique environmental (E) factors. Structural equation modeling, in the context of classical twin studies, builds on the notion that MZ pairs share 100% of their genetics and DZ pairs share, on average, 50%. Common environmental factors are assumed to be shared 100% by both MZ and DZ pairs.

We present parameter estimates, 95% confidence intervals, and goodness of fit statistics for 3 models: the full model (ACE), a model in which all variance was attributable to genetic and specific environmental factors (AE), and a model in which all variance was produced by common and specific environmental factors (CE). Reduced models were constructed by removing a specific parameter, and we compared the goodness of fit of each reduced model to the full model using a likelihood ratio χ^2^ test. Models were also evaluated using Akaike Information Criterion [24]. The model with the lowest Akaike Information Criterion was judged to be the best fitting and most parsimonious.

Descriptive statistics and correlations were computed using Stata 9.2 for Windows (StataCorp LP, 2006). Structural equation models were fit using MxGui version 1.4.06 (Department of Psychiatry, Virginia Commonwealth University, 2003). A significance level of 0.05 was considered criteria for a significant degradation of model fit.

## Results

We excluded 146 pairs due to our inability to determine zygosity (6%), 520 opposite-sex pairs (22%), and 357 pairs in which one or both twins were missing physical activity data (15%). The final sample consisted of 1,003 same-sex MZ and 386 DZ pairs. We compared select demographic characteristics in twins excluded from analyses due to missing physical activity data to those with valid physical activity data using t-tests. These groups did not differ in education, sex, or race. However, twins with valid physical activity data were significantly younger (∼5 years) than twins with missing data (P<0.01).

Select demographic and physical activity characteristics of same-sex twin pairs are presented in Table 1. This is a relatively young sample, with a mean age of 30 years. Further, the sample has a mean education of 14 years, is 62% female, and 85% non-Hispanic, White. Demographic characteristics were similar between MZ and DZ twin pairs, except that MZs were slightly younger (∼4 years) and had a lower proportion of pairs reporting non-Hispanic, White race/ethnicity (Ps<0.05). The average minutes of moderate-to-vigorous physical activity reported by MZ twins was higher than DZ (109 min/week vs. 99 min/week, P<0.05). Finally, 79% of MZ and 77% of DZ twins achieved the 60 minute per week activity threshold (P>0.05 for between-pair difference), whereas only 30% of MZ and 25% of DZ twins achieved the 150 minute per week activity threshold (P<0.05 for between-pair difference).

**Table 1:** Select characteristics of twins from same-sex pairs enrolled in the University of Washington Twin Registry.

Characteristic	Monozygotic	Dizygotic
	*n = *1,003	*n = *386
***Demographic***		
Age (Mean years±SD)	29±13*	33±15
Education (Mean years±SD)	14±2	14±2
Female (%)	61	63
Non-Hispanic, White (%)	84*	90
***Moderate-to-Vigorous Physical Activity***		
Total minutes per week (Mean minutes±SD)	109±76*	99±72
60-minute cut-point (%)	79	77
150-minute cut-point (%)	30*	25

* Indicates difference between monozygotic and dizygotic twin pairs at *P*<0.05; data for cut-points presented as percentage achieving that standard; SD, standard deviation.

**Figure 1:**
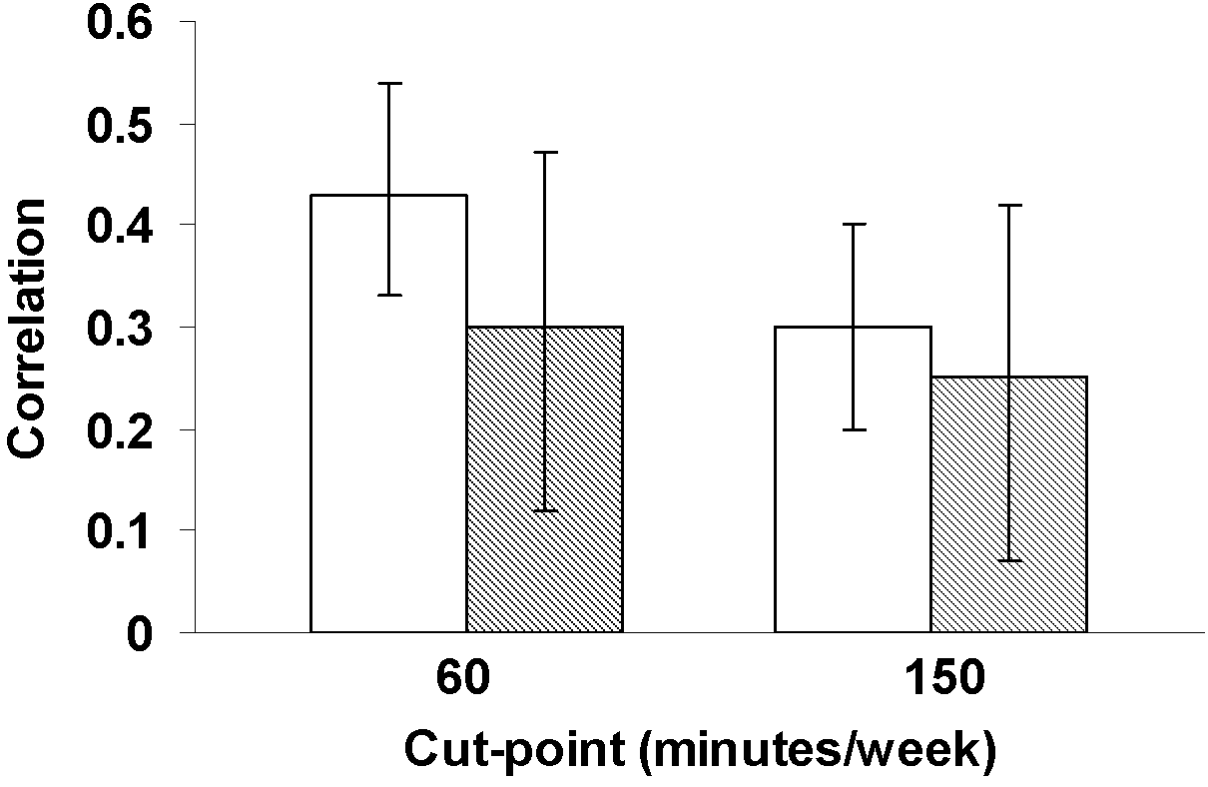
Within-pair tetrachoric correlations and 95% confidence intervals for each binary activity measure in monozygotic (solid white bars) and dizygotic (hatched bars) twins.

Within-pair comparisons using different cut-points are presented in Figure 1. Correlations were higher in MZ than DZ pairs using the 60 minute cut-point (r_MZ_ = 0.43 [0.33–0.54]; r_DZ_ = 0.30 [0.12–0.47]), suggesting genetic influence on physical activity participation using this standard. However, the correlations were attenuated using the 150-minute standard such that the strength of the association in MZ and DZ twins was similar in magnitude (r_MZ = _0.30 [0.20–0.40]; r_DZ = _0.25 [0.07–0.42]).

Using the lower cut-point, the best fitting model included additive genetics (A) and unique environment (E), with a heritability of 45% as shown in Table 2. In contrast, using the higher threshold, the best fitting model included the common (C) and unique environment (E), with the unique environment contributing 72% of the phenotypic variance.

**Table 2:** Univariate biometric genetic model for binary measures of physical activity in UWTR same-sex twin pairs.

Model*	Estimates of variance components†			Test of model fit			
	A	C	E	χ^2^	df	*P value*	AIC‡
***60 minute cut-point***							
ACE	0.27 (0.00, 0.53)	0.16 (0.00, 0.46)	0.57 (0.47, 0.67)	–	–	–	–
**AE**	**0.45 (0.34, 0.54)**	**–**	**0.55 (0.46, 0.66)**	**0.77**	**1**	**0.38**	**−1.23**
CE	–	0.39 (0.30, 0.48)	0.61 (0.52, 0.70)	1.76	1	0.18	−0.24
***150 minute cut-point***							
ACE	0.11 (0.00, 0.39)	0.18 (0.00, 0.36)	0.71 (0.61, 0.81)	–	–	–	–
AE	0.31 (0.21, 0.40)	–	0.69 (0.60, 0.79)	0.91	1	0.34	−1.09
**CE**	**–**	**0.28 (0.19, 0.36)**	**0.72 (0.64, 0.81)**	**0.31**	**1**	**0.58**	**−1.69**

* *ACE* refers to a model that includes additive genetics (A), common environment (C), and unique environment (E), *AE* only includes additive genetics and unique environment, and *CE* only includes common and unique environment;

† Proportion of variance (and 95% confidence interval) due to additive genetics, shared environment, and unique environment factors according to each model;

‡ Akaike's information criterion (AIC) is a global measure of goodness of fit, with the best-fitting and most parsimonious models shown in bold.

## Discussion

Our results suggest that unique environment factors provide the strongest influence on physical activity participation. This observation is contrary to findings reported by the GenomEUtwin collaboration, which suggested that genetic influences on physical activity participation were dominant. The major reason for this discrepancy is our use of the 150 minute per week activity threshold to categorize twins as exercisers. On the other hand, when we applied the 60 minute activity threshold used in the GenomEUtwin project [15], our heritability estimates were similar and supported an influence of genetic factors on physical activity levels. We found a heritability of 45% using the lower threshold, placing our estimate just below the range of 48–71% reported in most of the 7 countries in the GenomEUtwin project [15]. However, we found that the unique environment contributed most of the variance in physical activity participation even when using the lower standard.

Our findings underscore the importance of the threshold value used to define “exercisers” and “non-exercisers”. For example, a 60 minute per week cut-point might be useful in distinguishing individuals who are at least moderately active from those who are not regularly active and/or sedentary during an average week. The 150 minute per week standard likely distinguishes individuals who engage in regular, sustained activity (i.e., 30 minutes of moderate-to-vigorous activity per occasion on at least 5 days per week) from those who are not regularly active and/or sedentary on a regular basis. Individuals defined as exercisers using the higher activity threshold are more likely to derive greater health benefits, such as improved cardiorespiratory fitness, than those defined as exercisers using the lower threshold [25]–[27]. Many individuals categorized as exercisers using the 60-minute threshold, specifically those reporting between 60–150 minutes of activity per week, would not have met the minimum threshold of activity recommended for health improvements.

What is meant by “unique environmental factors”? In twin research, the common environment is defined as factors shared by members of a twin pair such as family, household, and neighborhood environments. Accordingly, the unique environment consists of factors that are not shared by members of a pair as they move to different environments. These unique environment factors include the built environment, defined as man made factors such as neighborhoods, sidewalks, streets, and parks that facilitate or promote engaging in physical activity. Interestingly, and consistent with the reports cited previously, we found common environment factors did not influence physical activity participation using the 60 minute standard but contributed 28% of the variance on physical activity participation using the 150-minute threshold. This observation suggests that household and family environment factors may also contribute to establishing long-term physical activity patterns.

As in most large epidemiologic studies, our physical activity data is based on self-report. However, our correlations and heritability estimates were consistent with those reported by the GenomEUtwin project when we used an identical cut-point to define exercise participation, thereby lending some credence to our measurement of physical activity. On the whole, monozygotic pairs were about four years younger than dizygotic twins. What effect this small statistical difference might have had on our results is unknown; however, any impact was likely very small. In addition, the generalizability of our data are limited because the sample is overwhelmingly non-Hispanic, White, and well educated. In forthcoming studies, we plan to explore the role of the unique environment specifically by obtaining objective measures of physical activity, food intake, and the built environment in a more diverse ethnic/racial mix of twin pairs.

### Conclusions

We observed little evidence of a genetic influence on physical activity. Instead, variation in physical activity was primarily due to common and unique environmental factors. Unique environmental factors contributed the greatest proportion of the variance to physical activity participation. This finding suggests that more efforts should be focused on determining what barriers to physical activity can be modified. Therefore, we suggest that obtaining indicators of the unique environment, such as measures of the built environment, is a productive avenue for future work. This type of research could help guide policy changes and have broad applications for improving public health.
